# The cellular machineries responsible for the division of endosymbiotic organelles

**DOI:** 10.1007/s10265-018-1050-9

**Published:** 2018-06-12

**Authors:** Yamato Yoshida

**Affiliations:** Department of Science, College of Science, Ibaraki University, Ibaraki, 310-8512 Japan

**Keywords:** Chloroplast division, Mitochondrial division, Endosymbiotic organelle, PDR1, MDR1

## Abstract

Chloroplasts (plastids) and mitochondria evolved from endosymbiotic bacteria. These organelles perform vital functions in photosynthetic eukaryotes, such as harvesting and converting energy for use in biological processes. Consistent with their evolutionary origins, plastids and mitochondria proliferate by the binary fission of pre-existing organelles. Here, I review the structures and functions of the supramolecular machineries driving plastid and mitochondrial division, which were discovered and first studied in the primitive red alga *Cyanidioschyzon merolae*. In the past decade, intact division machineries have been isolated from plastids and mitochondria and examined to investigate their underlying structure and molecular mechanisms. A series of studies has elucidated how these division machineries assemble and transform during the fission of these organelles, and which of the component proteins generate the motive force for their contraction. Plastid- and mitochondrial-division machineries have important similarities in their structures and mechanisms despite sharing no component proteins, implying that these division machineries evolved in parallel. The establishment of these division machineries might have enabled the host eukaryotic ancestor to permanently retain these endosymbiotic organelles by regulating their binary fission and the equal distribution of resources to daughter cells. These findings provide key insights into the establishment of endosymbiotic organelles and have opened new avenues of research into their evolution and mechanisms of proliferation.

## Introduction

Chloroplasts generate organic molecules and oxygen by photosynthesis (Jarvis and López-Juez 2013), a process which, over the past billion years, resulted in the greening of the Earth. Mitochondria generate the chemical energy needed for many biological processes in eukaryotic cells via the biosynthesis of adenosine triphosphate (Friedman and Nunnari 2014; Lane and Martin 2010). Plastids and mitochondria descended from endosymbiotic bacterial ancestors and possess their own genomic DNA.

These organelles are not produced de novo; their proliferation involves the binary division of pre-existing organelles; this division is carried out by specialized ring structures known as the plastid division machinery and the mitochondrial division machinery (Kuroiwa et al. 1998; Osteryoung and Nunnari 2003). These complex structures contain at least three types of rings at the constriction sites (Chen et al. 2018; Kuroiwa et al. 2008; Miyagishima et al. 2011; Osteryoung and Nunnari 2003). The plastid-dividing (PD) and the mitochondrion-dividing (MD) rings, which form the main framework of the division machinery, comprise ring-shaped bundles of nanofilaments on the cytosolic side of the outer envelope membrane of the corresponding organelle (Kuroiwa et al. 1998).

Although the proteins responsible for the assembly of the PD and MD rings have not been fully elucidated, our recent studies in the unicellular alga *Cyanidioschyzon merolae* revealed that the glycosyltransferase proteins PLASTID-DIVIDING RING1 (PDR1) and MITOCHONDRION-DIVIDING RING1 (MDR1) are involved in the assembly of the PD and MD rings, respectively (Yoshida et al. 2010, 2017). The FtsZ ring, which is composed of a homolog of the bacterial fission protein FtsZ, is a single ring that assembles at the division site beneath the inner membrane in the stromal region of the plastid or the matrix region of the mitochondrion (TerBush et al. 2013). FtsZ is a tubulin-like GTPase protein and can polymerize into a filament, in a similar fashion to tubulin hetero-polymerization (Yoshida et al. 2016). Whereas bacteria have one *FtsZ* gene in their genome, the plastid and mitochondrial *FtsZ* genes underwent duplication; therefore, a total of four *FtsZ* genes were present in the genome of the primitive photosynthetic eukaryotes (Matsuzaki et al. 2004; Miyagishima et al. 2004). The dynamin ring is a disconnected ring-like structure formed of a dynamin superfamily member protein on the cytosolic surface of the outer envelope membrane at the organelle division site (Miyagishima et al. 2003a; Osteryoung and Nunnari 2003). The large GTPase proteins Dnm2 (also known as DRP5B/ARC5 in *Arabidopsis thaliana*) and Dnm1 (also known as DRP1 in animals), which belong to the dynamin superfamily, were previously identified as essential factors for plastid and mitochondrial division, respectively (Bleazard et al. 1999; Gao et al. 2003; Miyagishima et al. 2003b; Nishida et al. 2003). Interestingly, these plastid- and mitochondrial-division genes are all encoded by nuclear genes, indicating that the division mechanisms of these organelles are strictly regulated by the host nucleus.

**Fig. 1:**
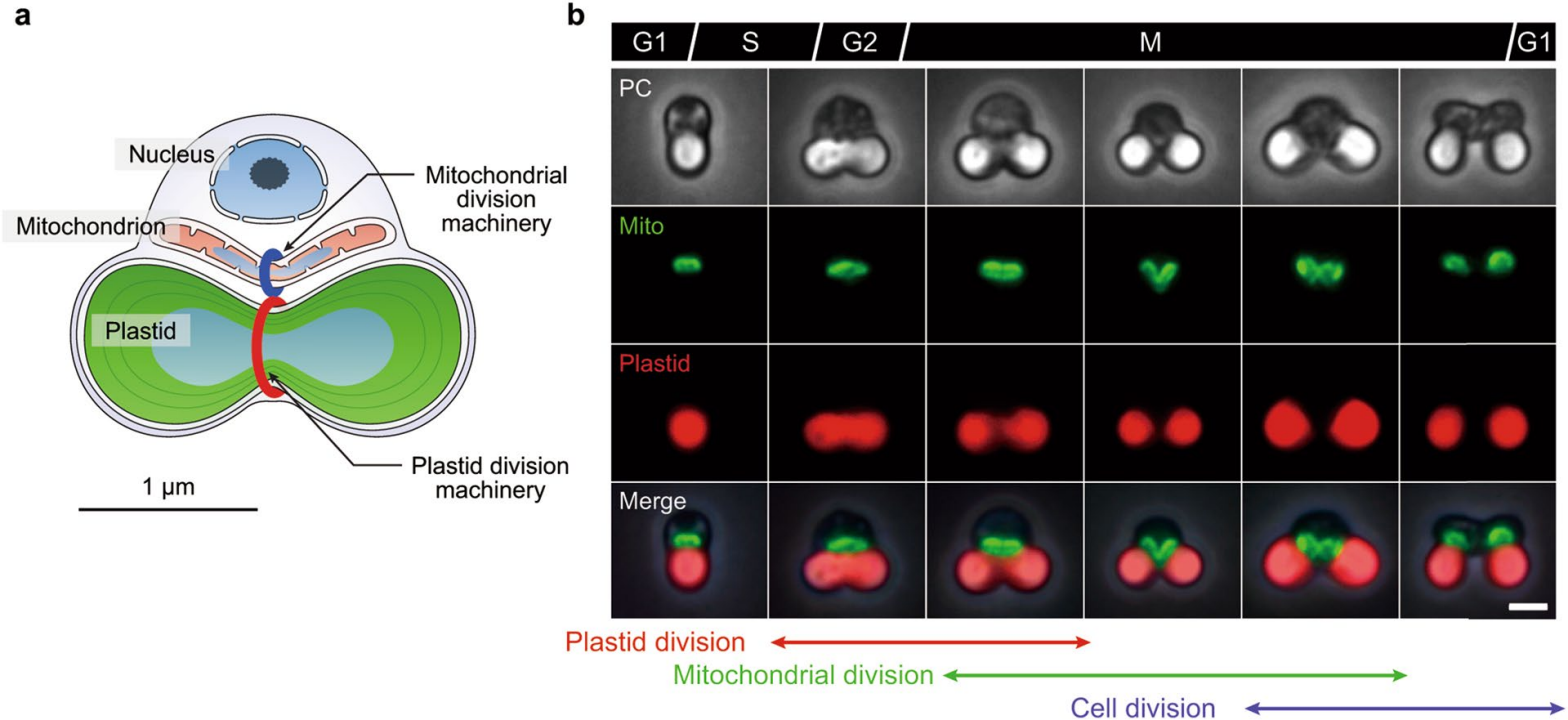
The primitive unicellular red alga *C. merolae*. **a** Schematic representation of a dividing *C. merolae* cell. **b** Sequential process of plastid, mitochondrial, and cell division in *C. merolae*. Mitochondria were imaged following immunostaining with an anti-mitochondrial-porin antibody, and the plastids were imaged by chlorophyll autofluorescence. PC, phase contrast. Scale bar, 1 µm. Images in **b** were reproduced and modified, with permission, from Yoshida et al. (2013)

To unveil the mechanisms of plastid and mitochondrial division, we previously studied the primitive alga *Cyanidioschyzon merolae* (Fig. 1a, b) (Kuroiwa 1998; Matsuzaki et al. 2004). Containing just one nucleus, one mitochondrion, and one plastid, the divisions of which can be highly synchronized, this alga offers striking advantages for the study of plastid and mitochondrial division. The simple genome of *C. merolae* also facilitates the use of various OMICS approaches. Over the past decade, my research group established methods for the isolation of the plastid- and mitochondrial-division machineries from *C. merolae* cells, enabling us to demonstrate that both are composed of an interconnecting complex of specialized ring structures on the organelle membranes (Yoshida et al. 2006, 2009, 2017). Furthermore, we identified several important components of these division machineries, and demonstrated that the outer and inner parts of the ring structures have different contractile properties to facilitate division (Yoshida 2018; Yoshida et al. 2012). Despite these discoveries, the underlying molecular mechanisms of plastid and mitochondrial division remain unclear, and many putative components of the division machineries are yet to be revealed. Fully elucidating the plastid- and mitochondrial-division mechanisms will likely require interdisciplinary approaches to help break the deadlock in this field. This review summarizes the current understanding of the structure and mechanisms of the plastid- and mitochondrial-division machineries, enabling the further exploration of the proliferation mechanisms in these important organelles.

## Structure and molecular dynamics of the plastid division machinery

**Fig. 2:**
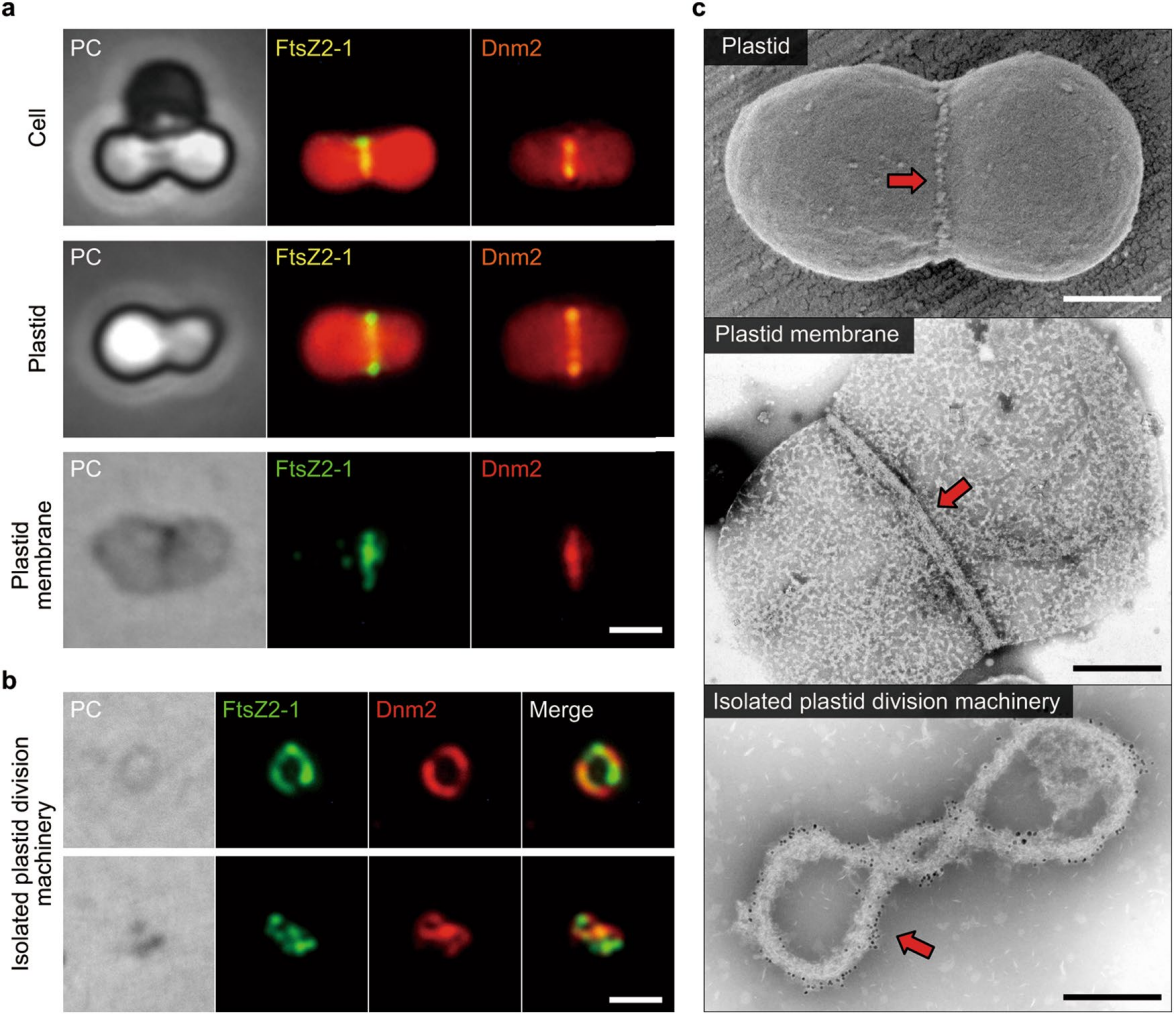
Isolation of plastid division machineries from dividing plastids in *C. merolae* cells. **a** Phase-contrast and immunofluorescence images of the FtsZ (FtsZ2-1: yellow/green) and dynamin (Dnm2: orange) rings of whole cells (top), isolated plastids (middle), and plastid membranes (bottom). Scale bar, 1 µm. **b** Isolated plastid division machineries. Scale bar, 1 µm. **c** Electron micrographs of a dividing plastid, a plastid membrane, and isolated plastid division machinery. Red arrows indicate plastid division machineries. Scale bars, 500 nm (top and middle) and 200 nm (bottom). Images were reproduced and modified, with permission, from Yoshida et al. (2006) and Yoshida et al. (2010)

The functional role of each ring in the plastid division machinery has been examined through investigation of the structure and mechanism of isolated intact plastid division machineries from synchronized *C. merolae* cells (Fig. 2a–c) (Yoshida et al. 2006). In addition to the circular rings, the isolated plastid division machineries formed super-twisted rings and spirals (Fig. 2b), which took both clockwise and counter-clockwise forms. When the plastid membranes were dissolved using a detergent treatment, the membrane-free plastid division machinery autonomously constricted. The existence of these topologically twisted structures and the autonomous constriction property indicated that the plastid division machineries themselves generate the contractile force for plastid division. The addition of guanosine triphosphate (GTP) did not stimulate any significant conformational changes of the division machineries; therefore, it is not clear whether FtsZ2 and Dnm2 GTPase activities are required for the generation of the contractile force. Longer treatments with the detergent resulted in FtsZ2- or Dnm2-free plastid division machineries. Many of the Dnm2-free plastid division machineries were straight, whereas the FtsZ2-free plastid division machineries could form spirals. These findings seemed to indicate the importance of the Dnm2 proteins in the contractile mechanism of the plastid division machinery; therefore, these machineries were further examined by optical trapping and manipulation experiments using optical tweezers. Individual spiral plastid division machineries stretched to approximately four times their original lengths returned to their original lengths upon release. In contrast, stretched Dnm2-free plastid division machineries were unable to recover their original lengths, indicating that the GTPase Dnm2 is likely to generate the motive force for contraction.

Examination of isolated *C. merolae* plastid division machineries using electron microscopy unveiled their nanoscale structure (Fig. 2c, bottom). A bundle of PD ring nanofilaments was clearly observed, each of which had an average width of 5–7 nm. Also, immuno-electron microscopy (immuno-EM) showed that FtsZ signals were located on the inner periphery of the circular PD ring when the membrane was dissolved. By contrast, Dnm2 proteins were located in a spiral along the outer periphery of the supertwisted and spiral-shaped plastid division machineries. Signals corresponding to Dnm2 were identified between the PD-ring filaments, suggesting that the Dnm2 molecules crosslink the PD ring filaments to generate the motive force for contraction by controlling the sliding movement of the filaments (Yoshida 2017; Yoshida et al. 2006).

## Elucidation of the molecular structure and assembly mechanism of the PD ring

In the 1980s and 1990s, electron microscopy observations revealed the presence of the PD ring at the division site of the plastids of numerous photosynthetic eukaryotes (Kuroiwa et al. 1998; Mita et al. 1986). Concurrently, it was shown that the PD ring is the main skeletal structure of the plastid division machinery and is universally present throughout the plant kingdom. The molecular components and assembly of the PD ring have been enigmatic, however. To elucidate the components of the PD ring, we digested isolated plastid division machineries with various proteases but could not decompose them. Furthermore, electron-dense deposits indicated that carbohydrates were present on the PD ring, suggesting that the PD ring is likely composed of saccharide molecules.

**Fig. 3:**
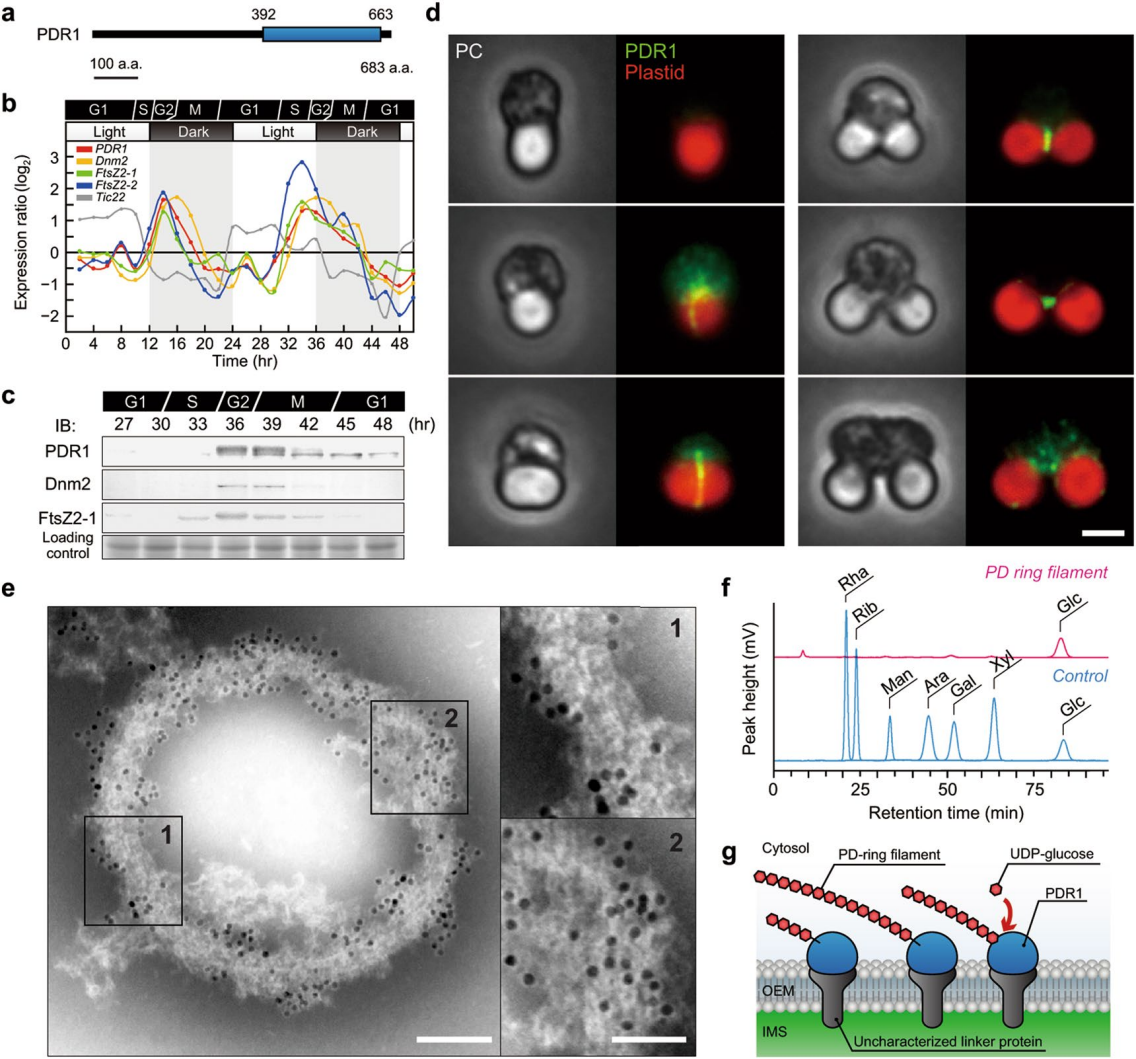
Identification of PLASTID-DIVIDING RING1 (PDR1) in *C. merolae*. **a** Domain architecture of CMR358C/PDR1. **b** Expression of *PDR1, Dnm2, FtsZ2-1*, and *FtsZ2-2* throughout the cell cycle, determined using a time-course transcriptome dataset from synchronized *C. merolae* cells (Fujiwara et al. 2009). **c** Protein levels of PDR1, Dnm2, and FtsZ2-1 throughout the cell cycle. **d** Immunofluorescence images of PDR1 in *C. merolae* cells. Scale bar, 1 µm. **e** Immuno-EM of PDR1 in the plastid division machinery. Many more PDR1 proteins were detected in the less-condensed regions of PD ring filaments than in the solid region (insets), suggesting that PDR1 protein molecules are associated with the whole PD ring filament, not just the surface. Scale bars, 100 nm (left) and 50 nm (right). **f** Fluorometric detection of the acid-hydrolysis products of the PD ring filament fraction using HPLC. **g** A schematic representation of PD ring filament biosynthesis by PDR1. Images were reproduced and modified, with permission, from Yoshida et al. (2010) and Yoshida (2018)

Based on this finding, we used a multi-omics approach in *C. merolae* to identify putative protein components of the PD ring that interact with the saccharide molecules. This led to the identification of an uncharacterized glycogenin-like protein, which was later designated PDR1 (Yoshida et al. 2010). A primary feature of PDR1 is a putative glycosyltransferase-8 (GT8) domain in the C-terminal region (Fig. 3a). Homologs of PDR1 have been widely identified in the photosynthetic eukaryotes, including in red algae and land plants (Yoshida et al. 2010). The *PDR1* mRNA and PDR1 protein levels specifically increased during the plastid division phase (Fig. 3b, c) (Fujiwara et al. 2009; Yoshida et al. 2010), and the immunofluorescence signals corresponding to PDR1 appeared earlier than Dnm2 in the cytoplasm, forming a ring from the beginning to the end of plastid division (Fig. 3d). Furthermore, knockdown of *PDR1* expression significantly decreased the frequency of plastid division, suggesting that this gene is essential for the fission of chloroplasts. Taking these observations together, we concluded that PDR1 is a novel plastid division protein.

We also investigated the interaction between PDR1 and the PD ring filament. PDR1 uniformly localized on the plastid division machineries (Fig. 3e), suggesting that PDR1 is a fundamental structural component of the division machinery. To investigate whether PDR1 binds to saccharide molecules, we looked for glycoproteins in the isolated plastid division machineries; this revealed that PDR1 is a glycoprotein. Componential analyses revealed that the purified PD ring filament fraction contained only glucose molecules (Fig. 3f). Taken together, these results show that the PD ring filaments are composed of PDR1-mediated polyglucan chains. PDR1 has a similar amino acid sequence to glycogenin, a priming protein for glycogen biosynthesis; therefore, PDR1 could function by elongating the glucan chains to biosynthesize PD ring filaments, analogous to the biosynthesis of glycogen (Fig. 3g) (Yoshida 2018; Yoshida et al. 2010).

## Structure and molecular dynamics of the mitochondrial division machinery

In the 1990s, an electron-dense specialized ring-like structure was found on the bridge of dividing mitochondria in the slime mold *Physarum polycephalum* (Kuroiwa et al. 1994, 1998); this structure was designated the MD ring. Electron microscopy observations revealed that the MD ring is assembled on the cytoplasmic side of the outer mitochondrial membrane (Kuroiwa et al. 1995, 1998, 2006). The MD ring was long believed to be the main structure driving mitochondrial division in primitive eukaryotes (Kuroiwa et al. 2006; Miyagishima et al. 2003a); however, the assembly mechanisms and molecular identity of the MD ring were only recently elucidated. The structural features and behavior of the MD ring are markedly similar to those of the PD ring, implying the involvement of similar molecular components. A method for the isolation of the mitochondrial division machinery from *C. merolae* cells was established, enabling further investigation of the MD ring. Nanoscale observations of the isolated mitochondrial division machinery showed that the MD ring is composed of a bundle of nanofilaments, each of which are 4–6 nm in diameter, in association with many additional proteins (Yoshida et al. 2017).

## Elucidation of the molecular identity of the MD ring and its evolutionary relationship with the PD ring

**Fig. 4:**
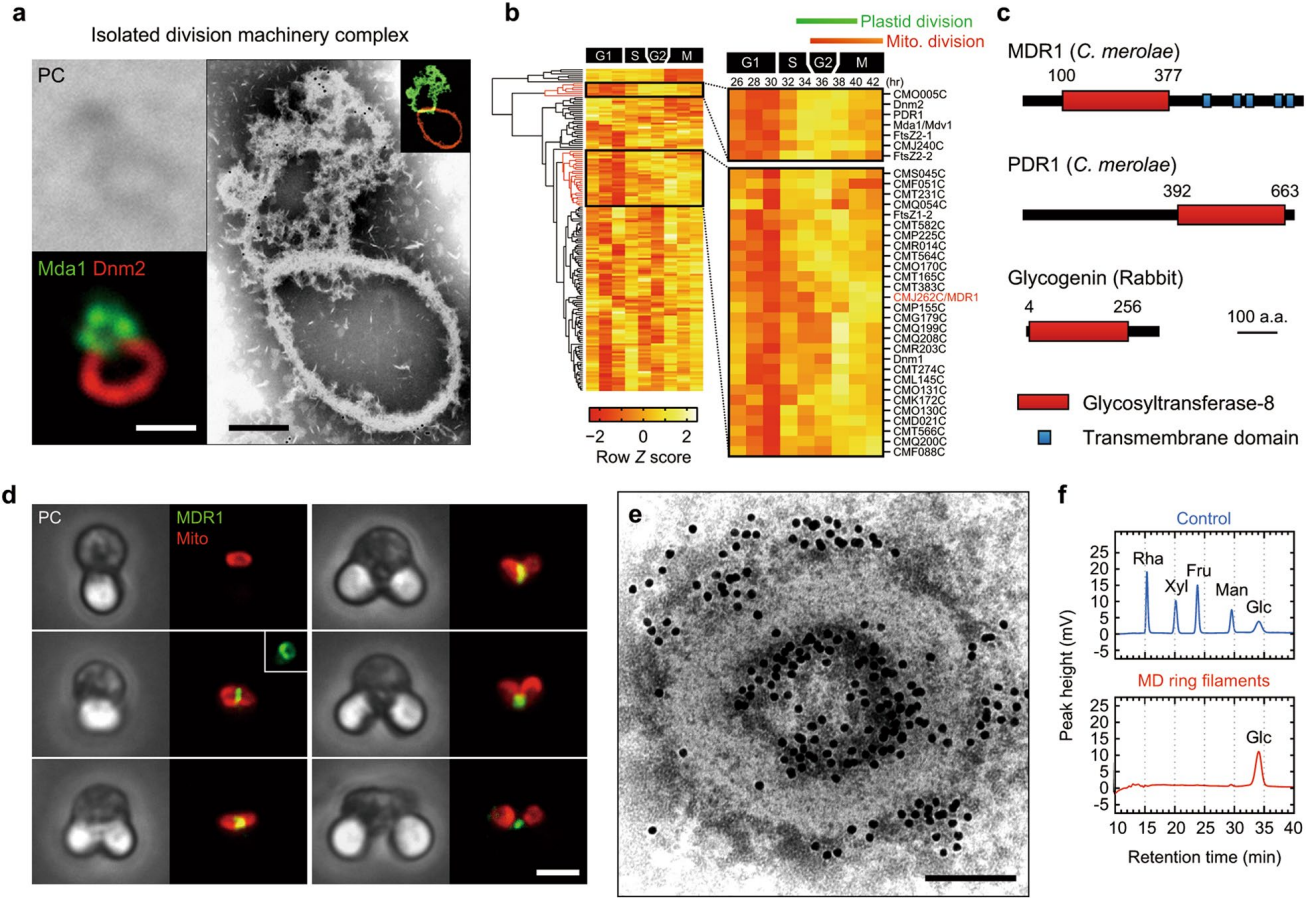
Identification of MITOCHONDRION-DIVIDING RING1 (MDR1) in *C. merolae*. **a** Isolated division machinery complexes containing the mitochondrial division machinery (green) and the plastid division machinery (red). Scale bars, 500 nm (left) and 200 nm (right). **b** Hierarchical clustering analysis of genes characterized using a proteomic analysis of isolated division machinery complexes. **c** Domain architecture of MDR1, PDR1, and glycogenin. **d** Phase-contrast and immunofluorescence images of MDR1. Scale bar, 1 µm. **e** Immuno-EM of isolated overdeveloped mitochondrial division machinery. Overdeveloped mitochondrial division machineries were isolated from mitochondrial-division-arrested *C. merolae* cells, which were obtained by treatment with a DNA synthesis inhibitor (see Yoshida et al. 2017 for more detail). In this image, 71% of the immunogold signals indicating the presence of MDR1 are located on the inner periphery region of the isolated mitochondrial division machinery. Scale bar, 100 nm. **f** Component analysis of the purified MD ring filaments using HPLC. Images were reproduced and modified, with permission, from Yoshida et al. (2017)

Using a modified technique for the isolation of the plastid division machinery, we can isolate the mitochondrial division machineries in a complex with the plastid division machineries (termed the division machinery complex) (Fig. 4a). A proteomic analysis of the division machinery complex fraction was combined with a time-course transcriptome dataset from synchronized *C. merolae* cells (Fujiwara et al. 2009) to enable the identification of candidate genes involved in mitochondrial division. Classification of these genes into groups using a hierarchical clustering analysis (Fig. 4b) showed that the genes in two specific groups had expression patterns indicative of a role in mitochondrial division. These genes included one that encodes a novel glycosyltransferase (CMJ262C), which was later designated MDR1 (Yoshida et al. 2017). The MDR1 protein contains a GT-8 domain in its N-terminal region and putative 5-repeat transmembrane domains in its C-terminal region (Fig. 4c). MDR1 homologs are found in various eukaryotes (Yoshida et al. 2017). The *MDR1* mRNA and its translated protein were specifically detected during the mitochondrial division phase, implying that MDR1 is a novel factor involved in mitochondrial division.

Immunofluorescence microscopy provided more concrete evidence of the involvement of MDR1 in mitochondrial division; the fluorescence signals corresponding to MDR1 were not detected in the cell during interphase, but the signals assembled into a single ring structure at the center of the mitochondrion just before mitochondrial division (Fig. 4d). The ring structure comprising MDR1 was retained at the division site during mitochondrial fission, indicating that MDR1 is very likely responsible for the contraction of the mitochondrial division site. The knockdown of *MDR1* expression using antisense suppression significantly reduced the frequency of successful mitochondrial divisions compared with the control cells, suggesting that MDR1 is essential for mitochondrial division. Furthermore, immuno-EM showed that the immunosignals corresponding to the MDR1 proteins were mainly detected in the inner periphery of the MD ring, with some detected on MD ring nanofilaments (Fig. 4e). Given that MDR1 contains putative 5-repeat transmembrane domains, these results suggest that MDR1 localizes along the division site on the outer mitochondrial membrane and is incorporated into the inner periphery region of the MD ring in vivo.

A componential analysis revealed that the only carbohydrate molecule within the purified MD ring filament fraction was glucose (Fig. 4f). Although the mechanism by which MDR1 biosynthesizes the MD ring filaments is still unclear, as is the mechanism by which PDR1 biosynthesizes the PD ring, an in silico analysis of the glycosyltransferase domain of MDR1 indicated that, like PDR1 and glycogenin, it also belongs to the GT-8 subfamily. Glycogenin catalyzes the biosynthesis of glycogen from a uridine diphosphate glucose donor substrate and belongs to the same subfamily as PDR1 and MDR1; therefore, we reasoned that MD ring biosynthesis may occur via a similar mechanism (Yoshida et al. 2017).

## Conclusions and remarks

A series of studies have shed light on the fundamental mechanisms by which plastid and mitochondrial division is achieved by their respective division machineries. In addition, a compelling structural and componential similarity between the plastid and mitochondrial division machineries was also demonstrated. Both division machineries consist of three rings, with the FtsZ ring inside the organelle and the PD/MD and dynamin rings on the cytosolic surface of the outer membrane. The plastid- and mitochondrial-division machineries are assembled by following processes. The FtsZ ring is assembled in the stromal region of the plastid or in the matrix region of the mitochondrion. Then, using the localization information, the specific glycosyltransferase proteins PDR1 or MDR1 are thought to bind to the outer membrane and biosynthesize the ring-shaped bundle of polyglucan nanofilaments known as the PD/MD ring. Subsequently, a GTPase dynamin-related protein, Dnm2 or Dnm1, is likely to crosslink the PD/MD ring filaments.

The assembled division machinery physically constricts the plastid/mitochondrial division site. Our studies indicated that an increased FtsZ protofilament turnover generates the contractile force in the FtsZ ring (Yoshida et al. 2016), while dynamin-related proteins crosslinking with the PD/MD ring filaments generate contractile forces by controlling the sliding movement of the PD/MD ring nanofilaments (Yoshida 2017; Yoshida et al. 2006). Taken together, these results suggest that the motive force for the fission of plastids and mitochondria is generated and regulated both inside and outside their respective membranes by contractible rings, the FtsZ ring and the PD/MD-dynamin ring, which form supramolecular protein nanomachines known as the division machineries.

**Fig. 5:**
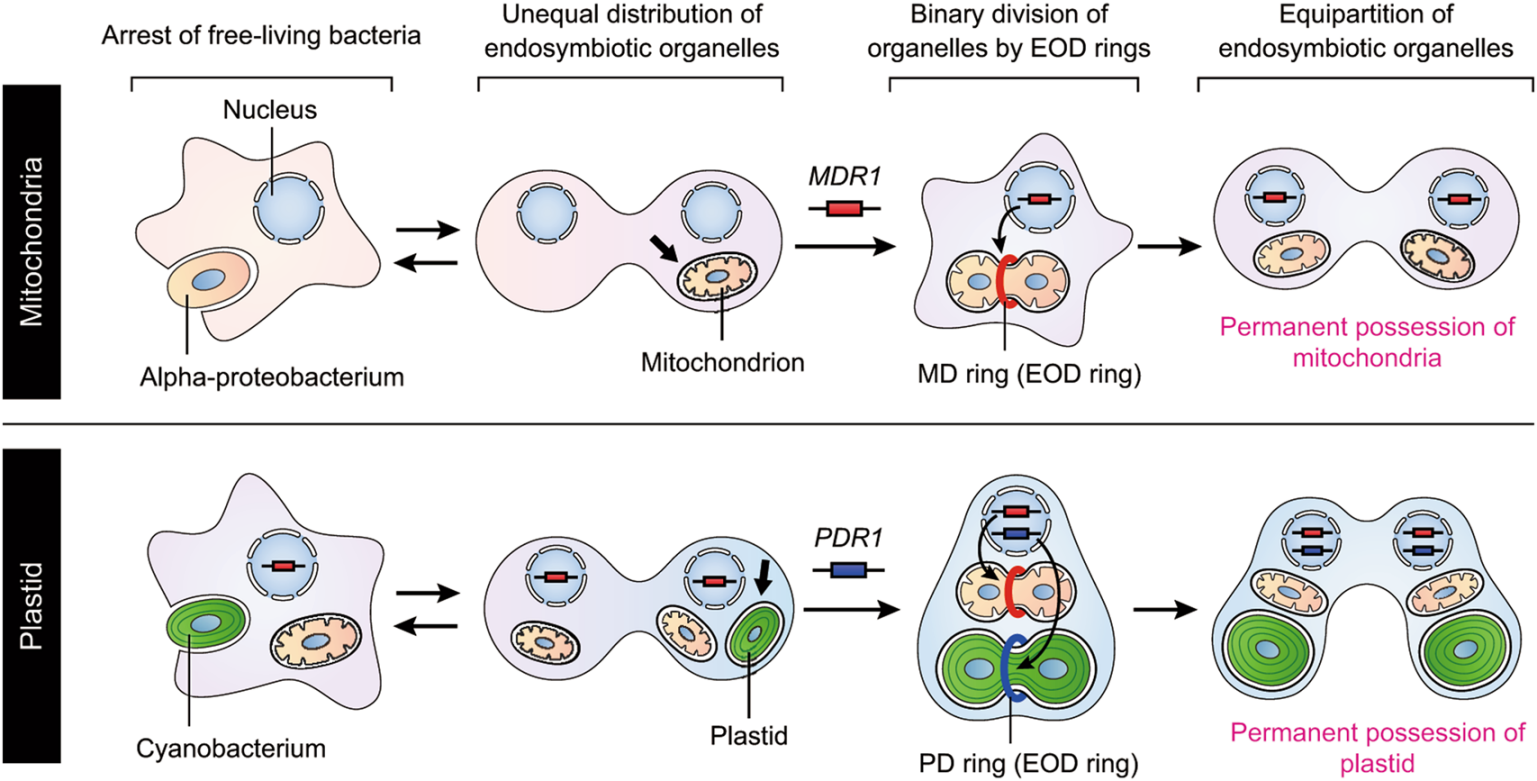
Proposed model for the evolutionary processes underlying the establishment of endosymbiotic organelles. See text and Yoshida et al. (2017) for more details. Schematic representations were reproduced and modified, with permission, from Yoshida et al. (2017)

Given that both mitochondria and plastids evolved from free-living bacterial ancestors, the significant similarities in structure and components between the MD and PD rings suggests that these structures were established in the host cells as endosymbiotic-organelle-dividing (EOD) rings, enabling the eukaryote to control the proliferation of its endosymbiotic organelles (Fig. 5). When the earliest endosymbiotic event occurred in the host eukaryotic ancestor, the endosymbiont (referred to as the α-proteobacteria) would not have divided and would have been transferred to one of the daughter cells during the division of the host eukaryote cell; therefore, it is likely that the host eukaryotic ancestor had to repeatedly capture the endosymbiont. The evolution of primitive division machinery comprising the MDR1-mediated EOD ring (MD ring) allowed the host cell to permanently possess mitochondria and distribute them to both daughter cells by regulating the binary fission of pre-existing mitochondria (Fig. 5, upper). Similarly, the plastid would have evolved from a cyanobacterial ancestor following the establishment of the division machinery comprising the PDR1-mediated EOD ring (PD ring) (Fig. 5, bottom). Given that MDR1 and PDR1 have homologous functions in mitochondrial and plastid division, respectively, in spite of their low sequence similarity, the appearance of the division machineries containing the EOD rings might have been a crucial, singular event enabling the emergence of endosymbiotic organelles (Yoshida 2018; Yoshida et al. 2017). Further investigation of MDR1 and PDR1 should provide insights into the mechanisms by which free-living α-proteobacteria and cyanobacteria evolved into the mitochondria and plastids, respectively, during evolution.
