# Correction to: The cellular machineries responsible for the division of endosymbiotic organelles

**DOI:** 10.1007/s10265-019-01093-y

**Published:** 2019-02-25

**Authors:** Yamato Yoshida

**Affiliations:** Department of Science, College of Science, Ibaraki University, Ibaraki, 310‑8512 Japan

## Correction to: Journal of Plant Research (2018) 131(5):727–734 10.1007/s10265-018-1050-9

The article The cellular machineries responsible for the division of endosymbiotic organelles, written by Yamato Yoshida was originally published electronically on the publisher’s internet portal (currently SpringerLink) on 12 June 2018 without open access.

With the author(s)’ decision to opt for Open Choice the copyright of the article changed on 25 February 2019 to © The Author(s) [Year] and the article is forthwith distributed under the terms of the Creative Commons Attribution 4.0 International License (http://creativecommons.org/licenses/by/4.0/), which permits use, duplication, adaptation, distribution and reproduction in any medium or format, as long as you give appropriate credit to the original author(s) and the source, provide a link to the Creative Commons license and indicate if changes were made.

The original article was corrected

